# Perception and determinants leading to antimicrobial (mis)use: A knowledge, attitude, and practices study in the rural communities of Odisha, India

**DOI:** 10.3389/fpubh.2022.1074154

**Published:** 2023-01-13

**Authors:** Matrujyoti Pattnaik, Ashish Kumar Nayak, Sonam Karna, Subrat Kumar Sahoo, Subrata Kumar Palo, Srikanta Kanungo, Jaya Singh Kshatri, Debaprasad Parai, Kamini Walia, Taru Singh, Hari Ram Choudhary, Sanghamitra Pati, Debdutta Bhattacharya

**Affiliations:** ^1^Department of Microbiology, ICMR-Regional Medical Research Centre (Department of Health Research, Ministry of Health and Family Welfare, Government of India), Bhubaneswar, Odisha, India; ^2^Division of Epidemiology and Communicable Diseases, Indian Council of Medical Research, New Delhi, India

**Keywords:** knowledge, attitude, practices, antibiotics, antimicrobial resistance

## Abstract

**Background:**

Antimicrobial resistance (AMR) has emerged as one of the major public health issues globally. This cross-sectional study determined knowledge, attitudes and practices (KAP) regarding antimicrobial use and AMR among rural communities of Tigiria (Odisha), India.

**Methods:**

A semi-structured questionnaire based on socio-demographic characteristics, antibiotics usage, awareness of antimicrobial resistance, healthcare utilization and quality of life were asked to the participants using an electronic device with Open Data Kit. Descriptive statistics, independent *t*-test and ANOVA were performed to analyze the variables.

**Results:**

A total of 1,003 participants were surveyed in the study from 25 villages of Tigiria. About 44.47% (95% CI: 41.36–47.60) of study participants have heard about antimicrobial medicines and 14.75% (95% CI: 12.65–17.13) of participants were involved in buying antibiotics without prescription over the counter. Around 20.14% (95% CI: 17.72–22.78) of participants, stopped taking antibiotics before completing the full course. The physical domain was the most affected with low scores compared to other domains of quality of life (QOL). The QOL scores were found significant (*p* < 0.05) across age, gender, education and ethnicity.

**Conclusion:**

The study documented a significant level of KAP regarding antimicrobial (mis)use in the study. It is essential that antimicrobial stewardship programs for various stakeholders and educational programmes must be initiated to increase awareness of people on antimicrobial resistance.

## 1. Introduction

The development of antibiotics has dramatically transformed medical care and resulted in a global decline in mortality and morbidity from infectious diseases (1). Due to the rise of antimicrobial resistance (AMR), the efficiency of currently used antimicrobial drugs is declining, making it more difficult and expensive to treat illnesses and harder to manage outbreaks. This has emerged as one of the major public health issues globally (2). Antimicrobial-resistant organisms (AMRO) have emerged globally as a result of microbial evolution, antimicrobial misuse and abuse in veterinary, human, and agricultural settings and poor infection control procedures, among other factors as key contributors to the global rise of AMR. This has resulted in difficult-to-treat infections, prolonged hospital stays, higher healthcare costs, and poor health outcomes (3, 4).

Developing nations in Asia and Africa are at greater risk of antimicrobial resistance (AMR), even though it's a matter of global concern. It is predicted that by 2050 over 10 million people would die from the disease worldwide including roughly 4 million people each from Asia and Africa, if no action is taken to stop the spread of AMR (5). The 68th World Health Assembly, which took place in May 2015, saw the adoption of a Global Action Plan (GAP) against AMR by its member nations (6). This strategy mandated that each nation create and carry out a National Action Plan for Antimicrobial Resistance (NAPAR) for combating AMR. The World Health Organization (WHO) is urging its member nations to adopt and put into practice the updated Model List of Essential Medicines, which divided antibiotics into the categories of Access, Watch, and Reserve (AWaRe) (7). The AWaRe categorization aims to increase access to antibiotics that can save lives and avoid the development of resistance from overuse of the selected priority medications.

In the Low and Middle-Income Countries (LMICs), a systematic study has revealed an increase of 77% in the rate of antibiotic consumption and an overall 114% rise in antibiotic consumption from 2000 to 2015 (8). During the year 2000–2010, a 76% rise in global antibiotic consumption was collectively contributed by BRICS countries, i.e., Brazil, Russia, India, China and South Africa (9). Unfortunately, India has one of the highest rates of antibiotic consumption with a massive 23% when compared to the other BRICS nations (9). According to a recent study in India, it is reported that the total defined daily doses (DDD) was 5,071 million in 2019 and the defined daily doses per 1,000 people per day (DID) was 10.4. Azithromycin was the most consumed antibiotic molecule (12.6%) followed by cefixime (10.2%) (10). As per the scoping report in 2017, more than 70% of the isolates of the Gram-negative bacteria, i.e., *Escherichia coli, Klebsiella pneumoniae*, and *Acinetobacter baumannii* as well as almost half of all *Pseudomonas aeruginosa* were resistant to fluoroquinolones and third generation cephalosporins. Gram-positive bacteria such as *Staphylococcus aureus* had a methicillin resistance rate of 42.6% while *Enterococcus faecium* had a vancomycin resistance rate of 10.5%. For ciprofloxacin, resistance rates among *Salmonella typhi* and *Shigella* species were 28% and 82%, respectively. Tetracycline resistance rates for *Vibrio cholerae* ranged from 17 to 75% in different parts of the country (11).

There is a lack of AMR research examining the complexity of upstream and downstream issues in a country like India, particularly in areas with limited resources. There is a need for generating evidence from well-designed studies from the community to execute policy development and implementation to prevent the irrational use of antibiotics and reduce the spread of AMR. The following study is a quantitative study that was conducted to map the knowledge relating to antibiotics, antibiotic resistance and antibiotic usage practices among the rural communities of Tigiria, Cuttack district of Odisha. Healthcare utilization and the quality of life from the study participants were also recorded during the survey.

## 2. Methodology

### 2.1. Study design

It is a cross-sectional study conducted to assess the knowledge, attitude and practices prevalent among rural communities regarding antibiotic usage and antimicrobial resistance in the state of Odisha, India. This study was conducted from November 2021 to January 2021.

### 2.2. Study settings

**Figure 1:**
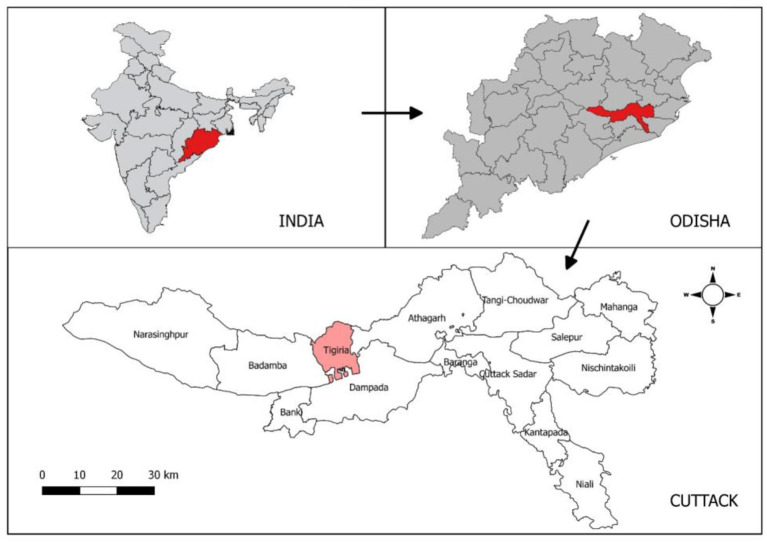
Study area site of the KAP in Odisha, India.

The study was undertaken in the rural settings of Tigiria block (“Block” is a district subdivision), Cuttack district which is situated in the coastal area of Odisha with 50 villages under it. Tigiria block is located at 20°29′0″N latitude and 85°31′0″E longitude. Tigiria has a tropical savanna type of climate where the average summer temperature is 45°C and the average winter temperature is 10°C. Tigiria receives 964 mm of rainfall annually. Average relative humidity varies from 55 to 79% throughout the year. The total population of the Tigiria block is 75,000 according to 2011 census. The study area map is given in Figure 1.

### 2.3. Sample size

The sample size was calculated using the formula (4pq/d^2^)^*^DE, where *p* = awareness of antibiotics, *q* = (1 – *p*), *d* = relative precision and DE = design effect. Assuming an awareness of antibiotics to be 50%, design effect of 1.3, relative precision of 4% and a non-response rate of 10%, the sample size was calculated to be 972 which was rounded off to 1,000.

### 2.4. Sampling method

Cluster sampling method was adopted for selecting study participants from the Tigiria block. There are 50 villages in Tigiria block out of which 25 villages were selected from the cluster sampling method. From each village, 40 households were selected for the survey. In each village, households were chosen through systematic random sampling method and only one adult individual was enrolled from each selected house. Simple random selection was used to choose a participant for the study if there were two or more adults living in a single household.

### 2.5. Data collection

An 86-item based questionnaire was developed to collect information from the study participants. The questionnaire consisted of semi-open questions based on socio-demographic characteristics, antibiotics usage, awareness of antimicrobial resistance, healthcare utilization and quality of life (WHO-QOL BREF scale) (12). The questionnaire was uploaded to electronic devices using an Open Data Kit. Questionnaire was developed in English and translated into the local language for a better understanding of the rural people. Hands-on training was provided to the field staff for collecting the data through electronic devices.

### 2.6. Data analysis

Data analysis was done using Statistical Package for Social Sciences (SPSS) version 21 (IBM^®^, USA). Distribution of frequency, percentages of variables and 95% confidence intervals (CI) were calculated for categorical variables. Continuous variables were calculated as mean with standard deviation (SD). Independent *t*-test and one-way analysis of variance (ANOVA) were used to determine the association of socio-demographic variables with WHOQOL-BREF domains. All tests of significance were two-tailed with a *p* < 0.05 indicating a statistically significant difference. Study area map was made using QGIS v3.10 which is an open-source software freely available on the internet.

### 2.7. Ethical concerns

Ethical clearance was obtained from the Institutional Human Ethics Committee of ICMR-Regional Medical Research Centre (RMRC), Bhubaneswar (ICMR-RMRCB/IHEC-2019/034) and the State Ethical Committee of Odisha. Each participant was explained about the aims and objectives of the study and provided with the participant information sheet for reference. Written informed consent was taken from every participant before taking part in the study.

## 3. Results

A total of 1003 participants were surveyed in the study and most of the participants were from the 18 to 45 years age group. The proportion of female participants was more than the male participants. Around 20.7% (*n* = 208) of the study population received no formal education, whereas 30.7% of them have completed schooling (Table 1). About 50% (*n* = 499) of the houses consist of household members around 1–4. More than 70% (*n* = 710) of the houses had nuclear family structure, followed by joint family (22.4%). About 59% of the study population belonged to other backward castes, followed by the general population (26.3%). Occupation of these study participants were as follows in increasing order of their proportion, housewife (38.2%), agriculture (24.5%) and business (12%).

**Table 1:** Socio-demographic characteristics of the rural study participants.

**Characteristics**	**Frequency (*n*)**	**Proportion (%)**
**Age**		
18–45	457	45.6
45–60	331	33.0
60 and above	215	21.4
**Gender**		
Male	447	44.6
Female	556	55.4
**Education**		
No formal education	208	20.7
Less than primary school	357	35.6
High school	308	30.7
Secondary school	41	4.1
College/pre-university	73	7.3
Post graduate degree	16	1.6
**Household size**		
1–4	499	49.8
5–8	435	43.4
9 and above	69	6.9
**Family type**		
Joint	225	22.4
Single	60	6.0
Nuclear	710	70.8
Extended	8	0.8
**Ethnicity**		
General	264	26.3
Schedule caste	121	12.1
Schedule tribe	26	2.6
Other backward castes	592	59.0
**Occupation**		
Agriculture	246	24.5
Business	120	12.0
Daily labor	86	8.6
Housewife	383	38.2
Unemployed	59	5.9
Private job	54	5.4
Govt. job	21	2.1
Student	34	3.4

Almost 44.47% (*n* = 446) of study participants have heard about antimicrobial medicines (Table 2). About 21.52% (*n* = 96) of participants knew about azithromycin and 9.41% (*n* = 42) about amoxicillin among the participants who have heard about antimicrobial medicines. About 28.03% (95% CI: 23.95–32.48) of participants took antibiotics for cold, 5.83% (95% CI: 3.91–8.53) for sore throat, 6.95% (95% CI: 4.84–9.82) for watery diarrhea, and 2.91% (95% CI: 1.63–5.06) for malaria. However, 14.75% (95% CI: 12.65–17.13) of participants were involved in buying antibiotics without prescription over-the-counter and 20.14% (95% CI: 17.72–22.78) of participants, stopped taking antibiotics before completing the full course. Similarly, 1.79% (95% CI: 1.09–2.87) of participants ask for antibiotics from health professionals on their own. Only 0.10% (95% CI: 0.005–0.06) of study participants knew about antimicrobial resistance.

**Table 2:** KAP on antimicrobial usage and AMR.

**Question**	**Response**	**Frequency (*n*)**	**Proportion (%)**	**95% CI**
Heard about a medicine called antimicrobial medicine/antibiotics (*n* = 1,003)	Yes	446	44.47	41.36–47.60
	No	557	55.53	52.39–58.63
Knowledge of antimicrobial medicines (*n* = 446)	Azithromycin	96	21.52	17.85–25.69
	Amoxicillin	42	9.41	6.94–12.60
	Cefixime	4	0.90	0.28–2.44
	Cefpodoxime	1	0.22	0.01–1.44
	Doxycycline	1	0.22	0.01–1.44
	Ampicillin	1	0.22	0.01–1.44
	Other medicines	55	12.33	9.43–15.83
	Don't remember	283	63.45	58.77–67.89
Antibiotics were consumed for what diseases (*n* = 446)	Cold	125	28.03	23.95–32.48
	Sore throat	26	5.83	3.91–8.53
	Watery diarrhea	31	6.95	4.84–9.82
	TB	2	0.45	0.07–1.79
	Malaria	13	2.91	1.63–5.06
	Don't remember	249	55.83	51.07–60.47
Heard of the problem of antimicrobial resistance (*n* = 1,003)	Yes	1	0.10	0.005–0.06
	No	1,002	99.90	99.35–99.99
Asked for the antibiotics to the health professional on your own (*n* = 1,003)	Yes	18	1.79	1.09–2.87
	No	995	99.20	98.36–99.62
Took antibiotics without a prescription over the counter (*n* = 1,003)	Yes	148	14.75	12.65–17.13
	No	855	88.23	86.04–90.13
Stopped taking antibiotics before completing the full course (*n* = 1,003)	Yes	202	20.14	17.72–22.78
	No	801	79.86	77.21–82.27

Participants were also asked about their healthcare utilization, and it was seen that 12.56% (*n* = 126) of the participants were sick during the last month of the interview (Table 3). Out of those 126 participants, 74 (58.73%) got sick once, 19 (15.08%) got twice and 33 (26.19%) got sick thrice in the last month. Participants had different ailments such as fever 64 (50.79%), weakness 70 (55.56%), body aches 57 (45.24%) and headaches 51 (40.48%). About 120 (95.24%) of the participants went for pre-hospitalization and 75 (62.50%) went to a government hospital/clinic for treatment.

**Table 3:** Healthcare utilization by the study participants.

**Question**	**Response**	**Frequency (*n*)**	**Proportion (%)**	**95% CI**
Sick for any time during the last 1 month (*n* = 1,003)	Yes	126	12.56	10.60–14.81
	No	877	87.44	85.18–89.39
Times you have been sick in the last 1 month (*n* = 126)	One	74	58.73	49.60–67.31
	Two	19	15.08	9.55–22.80
	Three or more	33	26.19	18.95–34.91
Your ailment each time (*n* = 126)	Fevers	64	50.79	41.78–59.75
	Weakness	70	55.56	46.45–64.31
	Body-aches	57	45.24	36.43–54.33
	Excessive fatigue and tiredness	15	11.90	7.04–19.17
	Headache	51	40.48	31.93–49.60
	Others	53	42.06	33.42–51.18
	Don't remember	7	5.56	2.45–11.52
Status of your ailment (*n* = 126)	Started more than 1 month ago and is continuing	32	25.40	18.25–34.06
	Started more than 1 month ago and has ended	32	25.40	18.25–34.06
	Started within 1 month and is continuing	23	18.25	12.15–26.34
	Started within 1 month and has ended	23	18.25	12.15–26.34
	Don't remember	16	12.70	7.65–20.09
Took any treatment for your illness (*n* = 126)	Yes	120	95.24	89.48–98.04
	No	6	4.76	2.04–10.30
Times, you have visited a health care provider for treatment (*n* = 126)	One	52	43.33	34.41–52.68
	Two	19	15.83	10.03–23.87
	Three or more	26	21.67	14.88–30.30
	Don't remember	23	19.17	12.77–27.57
Where did you go for treatment? (*n* = 126)	Government hospital/clinic	75	62.50	53.15–71.03
	Private hospital/clinic	26	21.67	14.88–30.30
	Pharmacist/dispensary	19	15.83	10.03–23.87
Accompanied you during each episode (*n* = 126)	None	30	25.00	17.74–33.88
	Spouse	38	31.67	23.64–40.87
	Son	24	20.00	13.47–28.49
	Daughter	1	0.83	0.04–5.23
	Son/daughter in-law	1	0.83	0.04–5.23
	Relatives	5	4.17	1.54–9.93
	Parents	5	4.17	1.54–9.93
	Don't remember	16	13.33	8.04–21.04
Who paid for your treatment? (*n* = 126)	Self	26	21.67	14.88–30.30
	Spouse	43	35.83	27.43–45.15
	Son	26	21.67	14.88–30.30
	Daughter	2	1.67	0.28–6.49
	Relatives	3	2.50	0.64–7.68
	Parents	8	6.67	3.13–13.12
	Don't remember	12	10.00	5.50–17.16
Reason for not taking any treatment (*n* = 6)	Facilities are available but lack of faith	1	16.67	0.87–63.51
	Financial reason	2	33.33	5.99–75.89
	Aliment not considered serious	1	16.67	0.87–63.51
	Others	2	33.33	5.99–75.89

The mean score across the four domains and the overall domain of WHOQOL-BREF based on sociodemographic factors was shown in Table 4. QOL of younger age participants (≤45 years) were observed with better scores significant (*p* < 0.05) in all their domains. Male participants had better QOL in physical, social and overall domains which were statistically significant (*p* < 0.05). With regard to education, illiterate participants had poor QOL in all domains when compared to educated participants (*p* < 0.05). Similarly, nuclear/single families displayed better QOL than joint families only in the social domain (*p* < 0.05). The QOL scores were found significant (*p* < 0.05) across ethnicity.

**Table 4:** WHO-QOL BREF scores based on socio-demographic variables.

	**Physical**	**Psychological**	**Social**	**Environmental**	**Overall**
	**Mean ±SD**	**Mean ±SD**	**Mean ±SD**	**Mean ±SD**	**Mean ±SD**
**Total**	46.35 ± 7.36	65.37 ± 10.17	71.58 ± 8.61	64.12 ± 9.31	61.86 ± 6.20
**Age**					
≤ 45 years	47.55 ± 6.66	67.69 ± 9.22	72.98 ± 6.35	65.25 ± 8.84	63.37 ± 5.18
>45 years	45.02 ± 7.86	62.81 ± 10.56	70.03 ± 10.35	62.86 ± 9.66	60.18 ± 6.78
*p*	< 0.001	< 0.001	< 0.001	< 0.001	< 0.001
**Gender**					
Male	47.14 ± 7.15	65.35 ± 9.86	72.36 ± 7.64	64.63 ± 8.56	62.37 ± 5.45
Female	45.71 ± 7.46	65.39 ± 10.42	70.94 ± 9.27	63.70 ± 9.85	61.43 ± 6.72
*p*	0.002	0.94	0.009	0.11	0.017
**Education**					
No formal education	43.13 ± 8.01	61.73 ± 11.01	69.13 ± 10.70	60.89 ± 10.33	58.72 ± 7.37
Received education	47.19 ± 6.94	66.33 ± 9.72	72.22 ± 7.86	64.96 ± 8.84	62.67 ± 5.58
*p*	< 0.001	< 0.001	< 0.001	< 0.001	< 0.001
**Occupation**					
Agriculture	46.07 ± 6.05	64.95 ± 9.51	72.51 ± 7.64	63.72 ± 8.82	61.81 ± 5.39
Housewife	46.45 ± 7.34	66.28 ± 10.73	71.40 ± 8.62	64.28 ± 9.60	62.10 ± 6.32
Others	46.43 ± 8.13	64.72 ± 9.95	71.14 ± 9.15	64.21 ± 9.33	61.63 ± 6.57
*p*	0.78	0.08	0.13	0.74	0.57
**Household size**					
1–4	46.15 ± 7.53	64.86 ± 9.88	71.39 ± 8.58	63.58 ± 9.51	61.49 ± 6.15
5 and above	46.55 ± 7.19	65.89 ± 10.43	71.76 ± 8.64	64.65 ± 9.08	62.21 ± 6.24
*p*	0.38	0.11	0.49	0.068	0.066
**Family type**					
Nuclear/single	46.52 ± 7.35	65.42 ± 9.58	72.10 ± 8.03	64.31 ± 8.68	62.09 ± 5.60
Joint	45.93 ± 7.37	65.27 ± 11.48	70.30 ± 9.76	63.63 ± 10.67	61.28 ± 7.42
*p*	0.25	0.83	0.003	0.28	0.06
**Ethnicity**					
General	46.80 ± 6.83	66.87 ± 9.79	72.18 ± 7.58	65.57 ± 8.63	62.85 ± 5.88
Other backward classes	46.05 ± 7.39	65.46 ± 10.34	71.34 ± 9.00	64.20 ± 9.45	61.76 ± 6.36
Schedule caste	46.30 ± 7.84	62.29 ± 9.79	71.26 ± 8.96	60.82 ± 9.54	60.17 ± 5.88
Schedule tribe	48.80 ± 9.01	62.53 ± 8.37	72.34 ± 7.74	62.61 ± 7.82	61.57 ± 5.49
*p*	0.18	< 0.001	0.55	< 0.001	0.001

## 4. Discussion

This study represents one of the attempts to assess the knowledge, attitude and practices of antimicrobial use and antimicrobial resistance in a rural setting in the eastern region of India. It is expected that the findings from the present study will provide baseline information related to understanding the use or misuse of antibiotics in rural Indian communities. However, there is insufficient knowledge about antimicrobial resistance in the rural community. The level of knowledge observed in our study about antimicrobials and their use is similar to the findings from Jordan and Nepal (13, 14). The lack of knowledge regarding antibiotic use and antimicrobial resistance is in accordance with the previous findings (13, 15). An increase in participants' education is an important criterion that depicts their knowledge gain about antibiotic use and antimicrobial resistance which was reported in the findings from earlier studies (13, 15–17). More than 50% of the participants were unaware of AMR similar to the finding reported in Nepal (14). The level of knowledge on AMR seen in our study, however, is lower than that reported in Norway (18). Very few studies have been done among the general population to determine their knowledge of AMR as seen in the literature. Studies have primarily focused on individuals or specific groups such as medical professionals, veterinary or students (17, 19, 20).

In the study, it was observed that participants have heard about antibiotics but as many of them have not received complete education, they were not able to tell the antibiotic medicines, they had used. Nearly one-seventh of the participants preferred taking antimicrobial medicines without a prescription over the counter or without consultation with healthcare workers. This was an unhealthy method of taking antibiotics in the rural population. Participants directly asking the health professionals to prescribe antibiotics was minimal. Very few people stopped taking the antibiotics before completing the full course which might be due to their compliant nature with the healthcare workers or for the fear of not getting completely cured. However, the knowledge of antimicrobial resistance in the rural community is almost negligible when compared with the other studies (13–16).

In the community, AMR is primarily caused by self-medication or inappropriate use of antimicrobials (21). However, in these settings, evidence-based antimicrobial stewardship programmes are very limited. Education must be a key element of antimicrobial stewardship programmes in order for them to be successful. Healthcare workers must be involved in antimicrobial stewardship programmes so that antibiotics can be preserved, AMR and substantial morbidity and mortality can be reduced. The distinctive legal and cultural traits of the study populations should be taken into account while designing intervention plans. The intervention can only be successful when both the healthcare workers and community work as a whole to reduce AMR.

Inadequate awareness of antibiotic use and AMR should be seen as worrying issues and possible signs of AMR that must be addressed right away. The data from this study might be used by policy- and decision-makers as one of the inputs to monitor drug usage policy and recommendations and reduce the risk of AMR. Our findings advocate for customized treatments, such as community-focused education campaigns about optimal antibiotic usage and AMR prevention strategies. For example, persons with less formal education, who have shown less understanding and poor practices about the use of antibiotics and steps to combat their resistance, should be the focus of education programmes.

The healthcare system was efficiently utilized by the study population across all age groups. It shows that the patients had better access to the healthcare system showing higher utilization in the rural area which might be due to multiple factors such as better transportation, etc. The physical domain of life analysis, which addressed working ability, daily activities, bodily discomfort, sleep and rest, mobility and energy received the lowest score as per the WHO-QOL BREF scale. The QOL scores in other domains were higher than the physical domain and were found to be significant across age, gender, education and ethnicity.

There are very few studies have been conducted on antimicrobial use and AMR in rural settings, especially in India which is one of the major advantages of our study. The sample size is also big and generalizable inferences can also be drawn from a similar setting. However, the study has some limitations such as the data might have been affected by recall bias or fear among rural study participants of not sharing undesirable practices. To draw causal inferences from this study is not possible as the study is cross-sectional in nature. Besides these limitations, the data from the study can provide baseline information that can help to carry out future research and track the efficiency of intervention studies.

## 5. Conclusion

This study provides information on knowledge, attitude and practices on antimicrobial usage and AMR from rural settings of Tigiria, Odisha. The study documented a low level of knowledge and a high level of practice on antimicrobial usage and AMR. The findings are crucial for directing policy development, programme planning, in execution of antimicrobial use and AMR-related initiatives focusing the community-based awareness, education, and sensitization.

## Data availability statement

The raw data supporting the conclusions of this article will be made available by the authors, without undue reservation.

## Ethics statement

The studies involving human participants were reviewed and approved by Institutional Ethical Committee, RMRC, Bhubaneswar. The participants provided their written informed consent to participate in this study.

## Author contributions

SPat, DB, KW, and TS were involved in the concept, planning, and formulation of the study. AN, SKar, SS, DP, and HC were involved in data collection. Data analysis and the initial draft were prepared by MP. SPal, SKan, and JK reviewed the manuscript. All the authors have read and approved the final version of the manuscript.
